# Impact of age on the diagnostic performances and cut-offs of APRI and FIB-4 for significant fibrosis and cirrhosis in chronic hepatitis B

**DOI:** 10.18632/oncotarget.17470

**Published:** 2017-04-27

**Authors:** Qiang Li, Chuan Lu, Weixia Li, Yuxian Huang, Liang Chen

**Affiliations:** ^1^ Department of Hepatitis, Shanghai Public Health Clinical Center, Fudan University, Shanghai 201508, China; ^2^ Department of Infectious Diseases, Huashan Hospital, Fudan University, Shanghai 200040, China

**Keywords:** chronic hepatitis B, liver fibrosis, cirrhosis, noninvasive marker, influence factors

## Abstract

**Aims:**

Assessing the diagnostic performances of APRI and FIB-4 using age as a categorical marker.

**Methods:**

822 chronic hepatitis B (CHB) patients were included. Using METAVIR scoring system as a reference, the performances of APRI and FIB-4 were compared between patients aged≥30 and patients aged<30 years.

**Results:**

The APRI AUROC in patients aged<30 years was lower than that in patients aged≥30 years for significant fibrosis (0.61 *vs* 0.70, *p*<0.001) and cirrhosis (0.64 *vs* 0.78, *p*<0.001). The FIB-4 AUROC in patients aged<30 years was lower than that in patients aged≥30 years for significant fibrosis (0.57 *vs* 0.65, *p*<0.001) and cirrhosis (0.63 *vs* 0.72, *p*<0.001). Using specificity≥90%, the APRI cut-off in patients aged<30 years was lower than patients aged≥30 years for significant fibrosis (1.0 *vs* 1.2) and cirrhosis (1.2 *vs* 1.5). Using sensitivity≥90%, the APRI cut-off in patients aged<30 years was also lower than patients aged≥30 years for significant fibrosis (0.2 *vs* 0.4) and cirrhosis (0.3 *vs* 0.5). Using specificity≥90%, the FIB-4 cut-off in patients aged<30 years was lower than that in patients aged≥30 years for significant fibrosis (1.2 *vs* 2.1) and cirrhosis (1.4 *vs* 2.6). Using sensitivity≥90%, the FIB-4 cut-off in patients aged<30 years was also lower than that in patients aged≥30 years for significant fibrosis (0.5 *vs* 0.8) and cirrhosis (0.8 *vs* 1.2).

**Conclusions:**

Evaluation of the diagnostic performances of APRI and FIB-4 should take age into consideration.

## INTRODUCTION

Globally, an estimated 240 million patients have chronic hepatitis B virus (HBV) infection, which is intermediate to high prevalence in Asia-Pacific region [1]. In China, the HBV seroepidemiology has already shown a decrease in the prevalence of HBsAg, from 9.75 % in 1992 to 7.18 % in 2006 [1, 2]. Chronic hepatitis B (CHB) patients with liver fibrosis were at increased risk of cirrhosis, and cirrhotic patients were at increased risk for liver de-compensation, hepatocellular carcinoma (HCC) and death [3]. A sustained suppression of HBV replication was associated with improvement in liver histology [4, 5]. According to CHB guidelines, patients with significant fibrosis or cirrhosis should receive antiviral therapy [1, 6–8]. Besides, evaluation of liver fibrosis has an important role in prognosticating patients and determination of candidacy for surveillance for HCC. Therefore, the assessment of liver fibrosis needs to be considered in patients in whom liver fibrosis or cirrhosis is suspected.

Liver biopsy is the gold standard to assess the degree of liver fibrosis, but limited by its high cost, invasiveness, and risk of complications [9]. Non-invasive fibrosis tests based on serum indices or ultrasound are increasingly used for evaluating liver fibrosis. The transient elastography performed with FibroScan is recognized as an excellent fibrosis test because of its high diagnostic performance, non-invasive procedure, and can be undertaken in outpatient [10]. However, the FibroScan is limited by the high cost of equipment and fee for maintenance [11]. Serum fibrosis indices such as the aspartate transaminase (AST) to platelet ratio index (APRI) and fibrosis index based on the 4 factors (FIB-4) consist of indirect markers such as alanine transaminase (ALT), AST and platelet count, which are associated with lower costs, do not require particular expertise in their interpretation, and can be performed in an outpatient setting [11]. Currently, APRI and FIB-4 have been used widely in clinical practice. However, one of the research gaps is to evaluate the impact of other factors on the diagnostic performances of APRI and FIB-4.

According to the American Association for the Study of Liver Diseases (AASLD) guidelines for the treatment of CHB, in patients who acquired HBV infection at birth or in early childhood, the average age of transitioning from immune-tolerant to immune-clearance phases is 30 years [6, 12]. According to the European Association for the Study of the Liver (EASL) guidelines for CHB, liver biopsy or even therapy should be considered in patients over 30 years of age and/or with a family history of HCC or cirrhosis [7]. According to the Asian-Pacific Asociation for the Study of the Liver (APASL) guidelines for CHB, assessment of liver histology is usually recommended to determine the stage of fibrosis in patients older than 30 years and with a high viral load [1]. In CHB patients, age over 30 years is associated with higher likelihood of liver fibrosis than those under 30 years [6, 13]. It was hypothesized that age might be an influence factor on the diagnostic performances of APRI and FIB-4. This study evaluated the impact of age on the diagnostic performances of APRI and FIB-4 in 822 CHB patients.

## RESULTS

### Baseline data

Baseline characteristics of enrolled patients were presented in Table 1. The majority of patients were male (517, 62.9%), HBeAg positive (576, 70.1%), and middle-aged (median 35 years). Median HBV DNA, ALT, AST, APRI, and FIB-4 was 6.5 log10 copies/ml (IQR=4.9–7.7), 44 IU/L (IQR=28–68), 32 IU/L (IQR=24–44), 0.48 (IQR=0.34–0.76), and 0.99 (IQR=0.69–1.50), respectively; and mean platelet count was 171×10^9^/L. Of 822 patients, 261 (31.8%) were classified as having significant fibrosis, and 85 (10.3%) having cirrhosis.

**Table 1:** Baseline characteristics of the study population

Characteristics	All patients n=822	Patients ≥30 years n=559	Patients < 30 years n=263	P value
Age (years)	35 (29-41)	39 (35-43)	26 (24-28)	**<0.001**
Male gender, n (%)	517 (62.9%)	355 (63.5%)	162 (61.6%)	0.597
HBeAg positive, n (%)	576 (70.1%)	405 (72.5%)	171 (65%)	**0.03**
HBVDNA (log10 copies/ml)	6.5 (4.9-7.7)	6.1 (4.6-7.7)	7.3 (5.5-7.8)	**<0.001**
ALT (IU/L)	44 (28-68)	43 (29-67)	47 (27-68)	0.866
AST (IU/L)	32 (24-44)	32 (25-45)	30 (24-42)	**0.043**
Platelet count (10^9^/L)	171 ± 51	168 ± 53	179 ± 46	**0.008**
APRI	0.48 (0.34-0.76)	0.51 (0.34-0.80)	0.41 (0.32-0.66)	**0.002**
FIB-4	0.99 (0.69-1.50)	1.15 (0.83-1.74)	0.68 (0.54-0.94)	**<0.001**
Liver fibrosis stage				
F0-1	561 (68.2%)	348 (62.3%)	213 (81.0%)	**<0.001**
F2-4	261 (31.8%)	211 (37.7%)	50 (19.0%)	**<0.001**
F3-4	145 (17.6%)	123 (22.0%)	22 (8.4%)	**<0.001**
F4	85 (10.3%)	74 (13.2%)	11 (4.2%)	**<0.001**

ALT, alanine transaminase; AST, aspartate transaminase; APRI, aspartate transaminase to platelet ratio index; FIB-4, fibrosis index based on the 4 factors; the parenthesis represents IQR

Of 822 patients, 559 (68%) had age≥30 years and 263 (32%) had age<30 years. Patients aged≥30 years had higher age (39 *vs* 26 years, *p*<0.001), proportion of HBeAg positive (72.5% *vs* 65%, *p*=0.03), AST (32 *vs* 30 IU/L, *p*=0.043), APRI (0.51 *vs* 0.41, *p*=0.002), and FIB-4 (1.15 *vs* 0.68,, *p*<0.001), but lower HBV DNA (6.1 *vs* 7.3 log10 copies/ml, *p*<0.001) and platelet count (168 *vs* 179 × 10^9^/L, *p*=0.008) than patients aged<30 years. Patients aged≥30 years had higher proportion of significant fibrosis (37.7% *vs* 19.0%, *p*<0.001) and cirrhosis (13.2% *vs* 4.2%, *p*<0.001) than patients aged<30 years.

### Correlation between noninvasive fibrosis tests and METAVIR fibrosis stages

**Figure 1:**
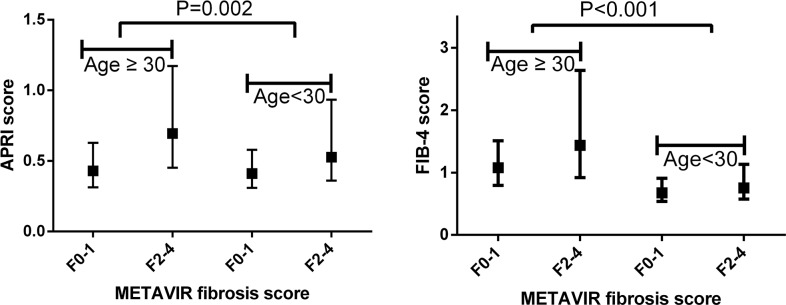
Association of noninvasive fibrosis tests with METAVIR fibrosis stages

The correlation of noninvasive fibrosis tests with METAVIR fibrosis stages was presented in Table 2 and Figure 1. In patients aged≥30 years, liver fibrosis correlated with APRI (r=0.36, *p*<0.001) and FIB-4 (r=0.29, *p*<0.001). In patients aged≥30 years, liver fibrosis correlated with APRI (r=0.16, *p*=0.011), but had no correlation with FIB-4 (r=0.07, *p*=0.295).

**Table 2:** Correlation between noninvasive fibrosis markers and METAVIR fibrosis stages

	Patients ≥30 years		Patients <30 years	
Variables	Spearman's r	P value	Spearman's r	P value
APRI	0.36	**<0.001**	0.16	**0.011**
FIB-4	0.29	**<0.001**	0.07	0.295

APRI, aspartate transaminase to platelet ratio index; FIB-4, fibrosis index based on the 4 factors

### Diagnostic performances of APRI and FIB-4 for significant fibrosis and cirrhosis

**Figure 2:**
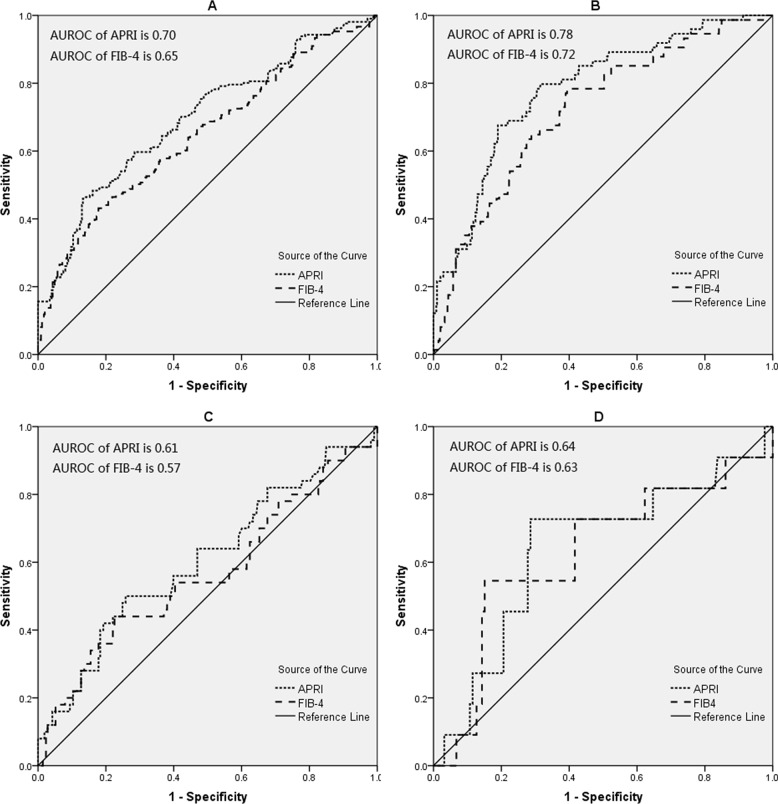
The ROC curves of APRI and FIB-4 for significant fibrosis and cirrhosis **(A)** for significant fibrosis in patients≥30 years; **(B)** for cirrhosis in patients≥30 years; **(C)** for significant fibrosis in patients≥30 years; **(D)** for cirrhosis in patients≥30 years.

In patients aged≥30 years, the area under the receiver operating characteristic curve (AUROC) of APRI was higher than FIB-4 to predict significant fibrosis (0.70 *vs* 0.65, *p*=0.004) and cirrhosis (0.78 *vs* 0.72, *p*=0.012) (Table 3 and Figure 2). In patients aged<30 years, the AUROC of APRI was comparable with FIB-4 to predict significant fibrosis (0.61 *vs* 0.57, *p*=0.11) and cirrhosis (0.64 *vs* 0.63, *p*=0.853) (Table 4 and Figure 2).

**Table 3:** AUROCs of APRI and FIB-4 for significant fibrosis and cirrhosis in patients≥30 years

	Significant fibrosis		Cirrhosis	
	AUROC	(95% CI)	AUROC	(95% CI)
APRI	0.70	(0.66-0.73)	0.78	(0.75-0.82)
FIB-4	0.65	(0.61-0.69)	0.72	(0.68-0.76)
Comparison of AUROC				
APRI *vs* FIB-4	*p*=0.004		*p*=0.012	

APRI, aspartate transaminase to platelet ratio index; FIB-4, fibrosis index based on the 4 factors; AUROC, the area under the receiver operating characteristic curve; CI, confidence interval.

**Table 4:** AUROCs of APRI and FIB-4 for significant fibrosis and cirrhosis in patients < 30 years

	Significant fibrosis		Cirrhosis	
	AUROC	(95% CI)	AUROC	(95% CI)
APRI	0.61	(0.54-0.67)	0.64	(0.58-0.70)
FIB-4	0.57	(0.50-0.63)	0.63	(0.57-0.69)
Comparison of AUROC				
APRI *vs* FIB-4	*p*=0.11		*p*=0.853	

APRI, aspartate transaminase to platelet ratio index; FIB-4, fibrosis index based on the 4 factors; AUROC, the area under the receiver operating characteristic curve; CI, confidence interval.

The AUROC of APRI in patients aged<30 years was lower than patients aged≥30 years for significant fibrosis (0.61 *vs* 0.70, *p*<0.001) and cirrhosis (0.64 *vs* 0.78, *p*<0.001). The AUROC of FIB-4 for patients aged<30 years was also lower than patients aged≥30 years for significant fibrosis (0.57 *vs* 0.65, *p*<0.001) and cirrhosis (0.63 *vs* 0.72, *p*<0.001) (Table 3 and Table 4).

### Diagnostic thresholds of APRI and FIB-4 in patients aged≥30 years

The cut-offs of APRI and FIB-4 for patients aged≥30 years were presented in Table 5. By obtaining a sensitivity of at least 90%, the low cut-off of APRI was 0.4 and 0.5, respectively; and the low cut-off of FIB-4 was 0.8 and 1.2, respectively, for significant fibrosis and cirrhosis. By obtaining a specificity of at least 90%, the high cut-off of APRI was 1.2 and 1.5, respectively; and the high cut-off of FIB-4 was 2.1 and 2.6, respectively, for significant fibrosis and cirrhosis.

**Table 5:** The cut-offs of APRI and FIB-4 for significant fibrosis and cirrhosis in patients≥30 years

Score	Classification	Cut-offs	Sensitivity, %	Specificity,%	PPV,%	NPV, %
APRI	Significant fibrosis	0.4*	91	24	42	81
		1.2**	32	90	66	69
	Cirrhosis	0.5*	91	36	18	96
		1.5**	32	90	33	90
FIB-4	Significant fibrosis	0.8*	90	20	41	77
		2.1**	31	90	66	68
	Cirrhosis	1.2*	91	32	17	96
		2.6**	35	90	35	90

APRI, aspartate transaminase to platelet ratio index; FIB-4, fibrosis index based on the 4 factors; PPV, positive predictive value; NPV, negative predictive value; Cut-offs* were established by obtaining a sensitivity of at least 90%; Cut-offs** were established by obtaining a specificity of at least 90%.

### Diagnostic thresholds of APRI and FIB-4 in patients aged<30 years

The cut-offs of APRI and FIB-4 for patients aged**<**30 years were presented in Table 6. By obtaining a sensitivity of at least 90%, the low cut-off of APRI was 0.2 and 0.3, respectively; and the low cut-off of FIB-4 was 0.5 and 0.8, respectively, for significant fibrosis and cirrhosis. By obtaining a specificity of at least 90%, the high cut-off of APRI was 1.0 and 1.2, respectively; and the high cut-off of FIB-4 was 1.2 and 1.4, respectively, for significant fibrosis and cirrhosis.

**Table 6:** The cut-offs of APRI and FIB-4 for significant fibrosis and cirrhosis in patients < 30 years

Score	Classification	Cut-offs	Sensitivity, %	Specificity,%	PPV,%	NPV, %
APRI	Significant fibrosis	0.2*	94	15	21	91
		1.0**	18	91	31	83
	Cirrhosis	0.3*	91	16	5	98
		1.2**	10	90	4	96
FIB-4	Significant fibrosis	0.5*	90	15	20	86
		1.2**	20	90	32	83
	Cirrhosis	0.8*	91	14	5	97
		1.4**	10	92	5	96

APRI, aspartate transaminase to platelet ratio index; FIB-4, fibrosis index based on the 4 factors; PPV, positive predictive value; NPV, negative predictive value; Cut-offs* were established by obtaining a sensitivity of at least 90%; Cut-offs** were established by obtaining a specificity of at least 90%.

## DISCUSSION

In this study, we evaluated the impact of age on the diagnostic performances and cut-offs of APRI and FIB-4. Using liver biopsy as a gold standard, the AUROC of APRI for patients aged<30 years was lower than patients aged≥30 years for significant fibrosis (0.61 *vs* 0.70, *p*<0.001) and cirrhosis (0.64 *vs* 0.78, *p*<0.001); and the AUROC of FIB-4 for patients aged<30 years was also lower than patients aged≥30 years for significant fibrosis (0.57 *vs* 0.65, *p*<0.001) and cirrhosis (0.63 *vs* 0.72, *p*<0.001). Thus it could be claimed that APRI and FIB-4 have better diagnostic performances for significant fibrosis and cirrhosis in CHB patients aged≥30 years, compared with patients aged<30 years.

In this study, APRI and FIB-4 use two cut-offs for diagnosing significant fibrosis or cirrhosis, as the use of a single cut-off would result in suboptimal sensitivity and specificity according to the recent WHO HBV guidelines [8]. A high cut-off with high specificity (i.e. fewer false-positive results) is used to diagnose patients with significant fibrosis or cirrhosis, and a low cut-off with high sensitivity (i.e. fewer false-negative results) to rule out the presence of significant fibrosis or cirrhosis. Using specificity≥90%, the high cut-off for APRI in patients aged<30 years was lower than that in patients aged≥30 years for significant fibrosis (1.0 *vs* 1.2) and cirrhosis (1.2 *vs* 1.5). Using sensitivity≥90%, the low cut-off for APRI in patients aged<30 years was also lower than that in patients aged≥30 years for significant fibrosis (0.2 *vs* 0.4) and cirrhosis (0.3 *vs* 0.5). Similar results were found in FIB-4. These results indicated that different cut-offs should be applied for APRI and FIB-4 based on patient age.

So far, several liver biopsy scoring systems have been developed, of which the METAVIR system, Knodell and Ishak scores are the most widely used [8]. Although the Ishak scoring system shows the necroinflammatory activity more clearly, the METAVIR scoring system was preferred in this study for the following reasons. First, according to the WHO HBV guideline, the diagnostic performances of APRI and FIB-4 for cirrhosis and significant fibrosis compared to METAVIR scoring system as the reference standard [8]. Second, according to the APASL guideline for CHB, APRI was recommended to diagnosis significant fibrosis (METAVIR≥F2) and cirrhosis (METAVIR=F4) using METAVIR scoring system as the reference standard [1]. Third, this study aimed to assess the diagnostic performances of APRI and FIB-4 for significant fibrosis and cirrhosis, rather than liver necroinflammatory activity.

In this study, 30 years was determined as a threshold value based on three reasons. First, age over 30 years is associated with higher likelihood of significant fibrosis and cirrhosis than those under 30 years in CHB patients [6, 13]. Second, patient who acquired HBV infection at birth or in early childhood, the average age of transitioning from immune-tolerant to immune-clearance phases is 30 years [6, 12]. In China, the majority of patients acquire HBV either at birth or early in childhood [14]. Third, all international guidelines for CHB recommended age over 30 years as one of the criterions for the assessment of liver histology to determine the stage of fibrosis [1, 6, 7].

Difference in cut-offs for APRI and FIB-4 between patients aged≥30 years and patients aged<30 years may be related to difference in prevalence of fibrosis, known as the spectrum bias [15, 16]. In this study, patients aged≥30 years had higher prevalence than patients aged<30 years for significant fibrosis and cirrhosis. Generally, the development of fibrosis is a step-by-step process starting from minimal fibrosis to cirrhosis, which may take years or decades. In patients without antiviral therapy, the longer the duration of HBV infection, the higher the likelihood for significant fibrosis, which indicated the duration of HBV infection associated with the development of fibrosis. Although it is difficult to get a precise duration of HBV infection in real-life situations due to the long non-symptom stage, we believe age is a surrogate marker of the duration of HBV infection in China where vertical transmission or infection in childhood was highly likely. Previous research has also shown that age was an independent predictor of significant fibrosis in CHB patients (OR=4.588, *p*=0.012) [17]. Similar results were showed in the study by Vardar et al, which found that age is associated with the extent of fibrosis [18].

However, several important caveats need to be noted. First, the PPV of all noninvasive fibrosis tests was low, especially for APRI and FIB-4, and many patients of significant fibrosis or cirrhosis will be missed using APRI or FIB-4 alone [8]. Therefore, it is important that APRI and FIB-4 are used alongside other clinical or laboratory criteria to identify significant fibrosis and cirrhosis. Second, the results of APRI or FIB-4 may be impacted by comorbidities, such as heavy alcohol intake (due to increase in AST), use of drugs (due to increase in ALT and AST), and malaria or HIV (due to decrease in platelet count) [8]. The impact of above conditions on the diagnostic performances of APRI and FIB-4 has not been fully evaluated. Last but not least, although APRI and FIB-4 are now commonly used, treatment decisions based on either false-positive or false-negative results need to be concerned. A false-positive result may lead to a patient being treated unnecessarily [8]. Conversely, a false-negative result means that a person with cirrhosis would not be identified, and may therefore not receive antiviral therapy [8].

There were several limitations in this study. First, the retrospective design might have caused selective bias [19]. Patients in this study had liver biopsy because of various clinical and laboratory indications such as age over 30 years, a family history of HCC or cirrhosis, a high HBV DNA load and fluctuant ALT level. Age over 30 years was one of the indications for liver biopsy, so the number of patients aged≥30 years was twice as many as patients aged<30 years in this study. Second, the prevalence of significant fibrosis and cirrhosis in this study might be higher than that at a community, because of patients in this study was based on a highly selected population who had liver biopsy because of various indications. Third, the detection limit of HBVDNA is 500 copies/ml, which is a very high value affecting the reliability of the study. Four, the number of F4 patients is markedly less than the number of F2-4 patients in both groups. A small number of cirrhotic patients may result in statistic bias and then affect the study results. Five, our study population, with high prevalence of HBeAg-positivity and narrow interval of years, might not be fully representative of CHB patients. The number of HBeAg (+) patients is large in this study. Although this is expected in the patient group aged<30 years, it seems a more-than-expected value in the patient group aged≥30 years.

In conclusion, APRI and FIB-4 as simple and practicable fibrosis index could identify patients with significant fibrosis or cirrhosis, and free a portion of CHB patients from liver biopsy. Different diagnostic performances and cut-offs were observed for APRI and FIB-4 between patients with age≥30 years and those with age<30 years, which indicated that more attention should be paid to the influence of age on the performances and cut-offs of noninvasive tests.

## PATIENTS AND METHODS

### Patients

Thirteen hundred and twenty-seven consecutive CHB patients who underwent liver biopsies in Shanghai Public Health Clinical Center, Shanghai, China between January 2010 and January 2017 were screened for inclusion. CHB was defined as the persistent presence of hepatitis B surface antigen (HBsAg) for more than six months [1]. Patients with following conditions were excluded: antiviral therapy (n=147); hepatitis C virus (HCV), hepatitis D virus (HDV) or human immunodeficiency virus (HIV) co-infection (n=87); alcohol consumption over 20g/day for more than 5 years (n=103); accompanied by nonalcoholic fatty liver disease (NAFLD) (n=128), or autoimmune liver disease (n=40). Finally, 822 patients were included.

All patients signed the informed consent before liver biopsy, and all clinical procedures were in accordance with the Helsinki declaration in 1983. The study protocol was permitted by the ethics committee of Shanghai Public Health Clinical Center.

### Liver histological examination

Ultrasonography-guided liver biopsy was performed under local anesthesia. Liver samples of minimum length 15mm were immediately 10% formalin-fixed and paraffin-embedded. Liver tissue with at least six portal tracts was considered sufficient for histologic scoring [20]. The METAVIR scoring system was adopted as the standard of liver fibrosis [21], which was classified into five stages: F0, no fibrosis; F1, portal fibrosis without septa; F2, portal fibrosis with rare septa; F3, numerous septa without cirrhosis; and F4, cirrhosis. All biopsy samples were interpreted independently by two liver pathologists who were blinded to non-invasive fibrosis tests. If they failed to reach an agreement, a third highly experienced pathologist reviewed the biopsy samples. In this study, we defined significant fibrosis as METAVIR F2-4, and cirrhosis as METAVIR F4.

### Blood fibrosis tests

The routine laboratory tests were performed the day before liver biopsies. The serological markers of HBV were detected with ELISA kits (Abbott, Wiesbaden, Germany). The HBV DNA was quantified by real-time PCR (Applied Biosystems, Foster City, USA), with the detection limit 500 copies/ml. The parameters including ALT and AST were measured by automation biochemistry analyzer (Hitachi, Tokyo, Japan). Platelet count was detected with automated hematology analyzer (Sysmex, Kobe, Japan). The calculation formulas of APRI and FIB-4 as follows: (1) APRI= (AST/ULN of AST)/platelet count×100; (2) FIB-4= (age × AST)/ (platelet count × (ALT)^1/2^).

### Statistics

Normality test of data was performed by Kolmogorov-Smirnov test. The baseline data was presented as follows: normal distribution data as mean ± standard deviation, non-normal distribution continuous data as median (interquartile range, IQR), and categorical data as number (percentage). Chi-square test (for categorical data), Mann Whitney test (for non-normal distribution continuous data), and t-test (for normal distribution data) was performed to identify statistical differences between two groups, respectively. The performances of APRI and FIB-4 were estimated using AUROCs [22]. The comparison of AUROCs was performed by MedCalc Statistical Software. APRI and FIB-4 use two cut-offs for diagnosing significant fibrosis and cirrhosis: (1) the low cut-offs obtaining a sensitivity of at least 90%; (2) the high cut-offs obtaining a specificity of at least 90%. Diagnostic accuracy was evaluated by sensitivity, specificity, positive predictive value (PPV), and negative predictive value (NPV). All significance tests were two-tailed, and *p*<0.05 was considered statistically significant. All statistical analyses were carried out using the SPSS statistical software version 15.0 (SPSS Inc. Chicago, Illinois, USA) and MedCalc Statistical Software version 16.1 (MedCalc Software bvba, Ostend, Belgium).

### Role of the sponsor

The funding organizations are public institutions and had no role in the design and conduct of the study; collection, management, and analysis of the data; or preparation, review, and approval of the manuscript.
